# ALKBH5 is a prognostic factor and promotes the angiogenesis of glioblastoma

**DOI:** 10.1038/s41598-024-51994-9

**Published:** 2024-01-14

**Authors:** Yugeng Fan, Dujuan Yan, Lijun Ma, Xiaoxi Liu, Guoqiang Luo, Yan Hu, Xin Kou

**Affiliations:** 1 Department of Neurosurgery, Yan’an People’s Hospital, Yan’an, China; 2 Xi’an New District Maternal and Child Health Care Institute, Xi’an, China; 3 The Affiliated Cardiovascular and Cerebrovascular Disease Hospital of Yan’an University, Yan’an, China; 4 Department of Neurosurgery, Tangdu Hospital, Air Force Military Medical University, Xi’an, China

**Keywords:** Cancer, Cell biology, Computational biology and bioinformatics

## Abstract

Despite numerous reports indicating the significant impact of RNA modification on malignant glioblastoma (GBM) cell behaviors such as proliferation, invasion and therapy efficacy, its specific involvement in glioblastoma (GBM) angiogenesis is remains unclear and is currently under investigation. In this study, we aimed to investigate the relevance between RNA modification regulators and GBM angiogenesis. Our study employed bioinformatic analyses, including Gene Set Enrichment Analysis (GSEA), differential expression analysis, and Kaplan–Meier survival analysis, to identify regulators of angiogenesis-associated RNA modification (RM). Gene Ontology (GO) and Kyoto Encyclopedia of Genes and Genomes (KEGG) analysis were applied to identify the enrichment of angiogenesis associated signatures in ALKBH5-high expression GBMs. We also utilized Western blot to verify the upregulation of ALKBH5 in clinical GBM samples. By a series of in vitro and in vivo assays, including plasmid transfection, wound healing, transwell invasion test, tube formation, RT-qPCR, ELISA assays and xenograft mice model, we validated the angiogenesis regulation ability of ALKBH5 in GBM. The N6-methyladenosine (m6A) modification “erase” ALKBH5 emerged as a candidate regulator associated with angiogenesis, demonstrating elevated expression and robust prognostic predictive ability in GBM patients. We also revealed enrichment of vasculature development biological process in GBMs with high ALKBH5 expression. Subsequently, we validated the elevated the expression of ALKBH5 in clinical GBM and paired adjacent tissues through western blot. Additionally, we knocked down the expression of ALKBH5 using sh-RNAs in U87 GBM cells to access the angiogenesis induction ability in U87 cells. In vitro experiments, Human Umbilical Vein Endothelial Cells (HUVECs) were used to perform wound healing, transwell migration and tube formation analysis, results indicated that ALKBH5 knock-down of U87 cells could decrease the pro-angiogenesis ability of U87 GBM cells. Further validation of our bioinformatic findings confirmed that ALKBH5 knockdown impaired VEGFA secretion in both in vitro and in vivo settings in U87 cells. These results comprehensively affirm the crucial role of ALKBH5 in regulating GBM-induced angiogenesis, both in vitro and in vivo. ALKBH5 not only emerges as a promising prognostic factor for GBM patients, but also plays a pivotal role in sustaining GBM progression by promoting angiogenesis.

## Introduction

Glioblastoma (GBM), the most lethal type of intracranial tumor, is characterized by highly infiltrative and aggressive growth, along with abundant neovascularization^1^. The current standard treatment for GBM involves surgical resection, chemotherapy and radiotherapy, a widely adopted approach for an extended period. Precision treatment plan, incorporating one or a combination of these therapies, are typically tailored for individual patients. However, the median overall survival (OS) time for GBM patients remains unsatisfactory despite these efforts^2,3^. Though great development has been achieved in cancer diagnosis and therapy in recent years, clinical advances in GBM treatment have been limited.. Recent reports suggest that anti-angiogenic reagents, such as bevacizumab, a type of monoclonal antibody of vascular endothelial growth factor (VEGF),may enhance the effectiveness of post-operative chemo-therapy and wreck recurrent GBMs. However, the role of RNA modification in anti-angiogenic therapy is still unveiled. Consequently, there is an urgent need to identify novel targets and develop new agents or inhibitors to advance GBM treatment^4^.

RNA modification represents a crucial facet of epigenetic regulation in eukaryotic cells, encompassing various modifications such as N6-methyladenosine (m6A), N7-methylguanosine (m7G), N1-methyladenosine (m1A), 5-methylcytidine (m5C), adenosine to inosine transition (I), pseudouridine (ψ), 5-methoxycarbonylmethyluridine (mcm5U) and 5-methoxycarbonylmethyl-2-thiouridine (mcm5s2U)^5^ among others. Notably, N6-methyladenosine (m6A) stands out as the most prevalent type of RNA modification in eukaryotic cells, with recent emphasis on its vital functions in regulating RNA stability, splicing, processing, translation and degradation^6–9^. The crucial role of RNA modification in cancers has garnered attention. exemplified by instances such as ALKBH5's modulation of cancer anti-PD-1 therapy efficacy by controlling lactate accumulation and suppressive cells in the cancer microenvironment^10^. Moreover, hepatitis B virus (HBV) infection has been linked to enhanced m6A modification of PTRN mRNA, impacting cancer immunity and contributing to the malignant progression of liver cancer^11^. Bioinformatic analysis have identified m6A-associated long non-coding RNAs (lncRNA) as promising prognostic biomarkers for WHO grade II–III gliomas^12^. Consequently, targeting m6A has emerged as a promising strategy for illuminating the path forward for cancer patients.

In our current study, our objective was to screen out the angiogenesis-associated RNA modification regulators from the 53 RM regulators. Employing a comprehensive set of bioinformatic analyses, including gene set enrichment analysis (GSEA), protein–protein interaction (PPI) analysis, Pearson correlation analysis, single-cell data analysis and Kaplan–Meier survival analysis, we pinpointed that ALKBH5 as a potentially significant and prognostic biomarker. Subsequent validation experiments confirmed that knockdown of ALKBH5 significantly impacted the angiogenesis promotion ability of glioblastoma (GBM) both in vitro and in vivo.

## Methods and materials

### Data acquisition

Transcriptomic and clinical data of GBM patients from the Cancer Genomic Atlas (TCGA) and the Chinese Glioma Genomic Atlas (CGGA) were downloaded from the UCSC dataset (https://xenabrowser.net/datapages/) and CGGA website (http://www.cgga.org.cn/), respectively. A total of 516 GBM samples were analyzed across three independent cohorts: TCGA-GBM (n = 145), CGGA-seq1 (n = 236) and CGGA-seq2 (n = 135). Transcriptomic data of normal human samples were sourced from the Genotype-Tissue Expression (GTEx) portal (https://gtexportal.org/home/). The genes encoding RNA modification regulators were acquired from a collection of prior study^5^.

### Gene set enrichment analysis (GSEA)

The gene set of “Angiogenesis” was fetched from the database of molecular signatures database (MSigDB, https://www.gsea-msigdb.org/gsea/msigdb/index.jsp). Before conducting GSEA analysis, TCGA-GBM patients were divided into two subgroups based on the top 30% and bottom 30% expression of each RNA modification regulator (RM). Differential expression analysis (DEA) was subsequently conducted to identify differential expression genes (DEGs) associated with each RM regulator expression via the R packages “Simpleaffy”^13^ and “affy”^14^. The criteria for DEGs were set at log2 (fold change) > 1 and *p* value < 0.05. Subsequently, GSEA analysis was conducted via utilizing the R package “clusterProfiler”^15^. The most positive and negative associated RM regulators were recognized based on the criteria: normalized enrichment score (NES) > 1.9 or < − 1.9, and normalized *p* value < 0.05.

### Protein–protein interaction (PPI)

The data of protein–protein interaction (PPI) was obtained from the website of STRING (https://string-db.org/). A total of 36 angiogenesis regulators downloaded from the MSigDB, fiftly-two RM regulators obtained from the previous publication were co-input into the software of Cytoscape (version 3.7.1). Both data analysis and figure visualization were performed within Cytoscape.

### Pearson correlation analysis

Pearson correlation analysis was employed to assess the statistical correlations between the expressions of angiogenesis regulators and RNA modification regulators. Pearson Correlation coefficient “R value” and statistical *p* value were calculated to evaluate the correlation level and significance between two factors.

### Expression comparison analysis

The most positive and negative enriched RM genes were used to conduct expression comparison between normal brain tissues (NBTs) and TCGA GBMs. Willcoxon’s rank sum test was applied to compare the expression levels between them. Statistical *p* value < 0.05 was the threshold to distinguish the statistical significance.

### Single cell data research

Single-cell data of GBMs were sourced from a previous study^16^ and analyzed in the Tumor Immune Single-cell Hub (TISCH, http://tisch.comp-genomics.org/^17^. The analysis procedure was conducted according to previous studies^18^. The data, identified by the serial number GSE89567 in the Gene Expression Omnibus (GEO, https://www.ncbi.nlm.nih.gov/gds), encompassed 6341 cells from 10 GBM patients, categorized into AC-like Malignant, Mono/Macro, OC-like Malignant, and Oligodendrocyte cell types. Scatter diagrams were generated to illustrate the distribution of RM regulators across these cell types.

### Kaplan–Meier survival analysis

Kaplan–Meier survival model was applied to judge the prognostic role of RM regulators across three independent GBM cohorts. The R packages “survival” (version 3.3–1) and “survminer” (version 0.4.9) were used to analyze and visualize the survival curves. Statistical *p* values were calculated by the method or log-rank test. *p* < 0.05 was set as statistical significance standard. The ‘surv-cutpoint’ function of R package “survminer” was utilized to select the most statistically significant cut-off for each analysis.

### Human protein atlas (HPA) and protein interaction analysis

Immunofluorescent staining figures of ALKBH5 in U251 GBM cell line and the immunohistochemical staining data of glioma samples with low- and high grade were obtained from the Human Protein Atlas (HPA, https://www.proteinatlas.org/^19^.

### Interactors of ALKBH5 protein

Information of ALKBH5 interactors were downloaded from the Compartmentalized Protein–Protein Interaction Database (ComPPI, version 2.1, https://comppi.linkgroup.hu/). The protein codes were mapped and translated in the Uniport database (https://www.uniprot.org/). Protein–protein interactions were visualized by the R package “ggplot2”.

### Sample collection

Seven paired GBM and adjacent tissues were collected from the inpatients in the department of neurosurgery of the Yan’an People’s Hospital from 2020 to 2021. All tissues were immediately stored in the liquid nitrogen after resection. Patients involved in this study had provided informed consent for the usage of sample, and the usage of clinical samples was in strict accordance with the guideline of the Medical Ethics Committee of Yan’an People’s Hospital.

### Gene ontology and encyclopedia of genes and genomes analysis

GBM patients in the TCGA cohort were grouping as the description in the “Gene Set Enrichment Analysis” part. Differential expressed genes (DEGs) were identified by R package “limma”^20^ under the standard of |log2(fold change) |> = 1 and *p* value < 0.01. Then the DEGs were input to perform the GO and KEGG analysis using R package “clusterProfiler”^15^, the top fifteen terms in each project, such as biological process (BP), molecular function (MF), cellular component (CC) and KEGG pathway were visualized in the bubble plots.

### Cell lines and cell culture

The U87 GBM cell line and Human Umbilical Vein Endothelial Cells (HUVECs) were purchased from the American Type Culture Collection (ATCC, USA). Dulbecco's modified eagle medium (DMEM, Gibco) and Endothelial Cell Mediums (ECM, ScienCell) were used to culture U87 and HUVEC cells, respectively. Except 5% fetal bovine serum (FBS, Gibco) and 1% Penicillin/Streptomycin solution (P/S, Gibco) were added in both mediums, 1% Endothelial Cell Growth Supplement (ECGS, ScienCell) was used to maintain HUVEC culture. 10% FBS and 1% Penicillin/Streptomycin solution were supplemented in DMEM for culturing U87 cells. Both cells were grown in 5% CO_2_ at 37 °C conditions.

### Short hairpin RNA (shRNA) construction and transfection

To achieve ALKBH5 knockdown, U87 cells were transfected with lentivirus packaging sh-RNAs targeting ALKBH5, using the pLKO.1—TRC cloning vector from the addgene (#10878, addgene, USA). The specific shRNA sequences of targets are listed: shALKBH5-1: 5′-GACTCTTGATGACCGCGTT-3′; shALKBH5-2: 5′-GAAGCTTCAATGGTCTCCTTA-3′; and a non-targeting control (sh-Con): 5′-TTCTCCGAACGTGTCACGT-3′. Transfection reagent Lipofectamine 3000 (#L3000008, ThermoFisher Scientific, USA) was employed to sensibilize the cell transfection reactions.

### RNA isolation and reverse transcription quantitative polymerase chain reaction (RT-qPCR)

Cell total RNA was extracted by using RNA isolation kit (S1550S, New England Biolabs, USA) under the instruction of protocol. Subsequently, 100 ng of total mRNA was reverse transcribed using TaqManTM universal master mix II (#4440042, ThermoFisher Scientific, USA) according to the manufacturer’s protocol. The ALKBH5 mRNA expression level was quantified from amplified cDNA, and normalized by the GAPDH expression, which was set as internal control. The ΔΔCT method was utilized to compared the expressions of ALKBH5 a different groups. One-way ANOVA was the statistical method for calculating the significance of distinction. The primers used in our study were listed below: ALKBH5 Forward: 5′-CCCGAGGGCTTCGTCAACA-3′, Reverse: 5′-CGACACCCGAATAGGCTTGA-3′; VEGFA Forward: 5′-GGGCAGAATCATCAC GAAGT-3′, Reverse: 5′-TGGTGATGTTGGACTCCTCA-3′; FGFR1 Forward: 5′-GTGGCT-GTGAAGATGTTGAA-3′, Reverse: 5′-GCC-AGGTCTCGGTGTATGCA-3′; VAV2 Forward: 5′-TCAGGCCTTTTCCCTCAGAG-3′, Reverse: 5′-TGCACCTCCACCTTGATGAT-3′; GAPDH Forward: 5′-ACCCAGAAGACTGTGGATGG-3′, Reverse: 5′-CAGTGAGCTTCCCGTTCAG-3′.

### Protein extraction and western blot

Cell lysis buffer (#R0010, Solarbio, China) was used to collapse the cells on the ice, phenylmethanesulfonylfluoride (PMSF, #P0100, Solarbio) was added in one hundredth of the volume of lysis buffer to restrain the degradation of protein. The protein concentration of lysates was quantified using the Enhanced BCA Protein Assay Kit (#P0010S, Beyotime, China). The western blot assay was strictly following the procedures of a previous publication^21^. The primary antibodies included ALKBH5 Monoclonal antibody (#67811-1-Ig, Proteintech, China), VEGFA Monoclonal antibody (#66828-1-Ig, Proteintech, China), VAV1 Polyclonal antibody (#16364-1-AP, Proteintech, China), FGFR1 Monoclonal antibody (#60325-1-Ig, Proteintech, China), and GAPDH Polyclonal antibody (#10494-1-AP, Proteintech, China). The horseradish peroxidase (HRP)-conjugated affinipure goat anti-rabbit IgG (1:5000, SA00001-2, Proteintech, China) and HRP-conjugated affinipure goat anti-mouse IgG (1:5000, SA00001-1, Proteintech, China) served as corresponding secondary antibodies after incubation with primary antibody. The value of protein was calculated using the ImageJ software.

### Enzyme‑linked immunosorbent assay (ELISA)

The quantification of human-derived VEGFA secretion from U87 cell lines was conducted using the enzyme-linked immunosorbent assay (ELISA) kit from Proteintech (#KE00216, China). The procedure was applied in strict accordance with the manufacturer’s protocol provided by the kit.

### Wound healing and transwell assay

For the wound healing assay, a total of 10^5 HUVEC cells were seeded in a well of 6-well plates prior to the assay. Once the cells firmly attached in the well, a straight line was scratched into the fully confluent monolayer using a 1 ml tip. The cultured medium of U87 cells (control and sh-ALKBH5 transfected) were then added to replace the ECM for 24 h culture. Pictures were captured at 0 h and 24 h timepoint after medium replace.

In the transwell assay, a total of 20,000 HUVEC cells were placed in the transwell chamber (# 07-200-150, Corning, USA), and an equal number of GBM cells were seeded in the wells of 48-well plates. After 24 h co-culturing, the wells were washed three times using PBS, and stained the cells using gentian violet solution (#G1072, Solarbio, Beijing, China) for 1 h. Subsequently, the cells were washed,, and photographed the cells by using microscope.

### Tube formation assay

To explore the role of GBM intrinsic ALKBH5 in tumor angiogenesis, the culture medium of U87 cells (control and sh-ALKBH5 transfected) were collected to dilute Matrigel with 1:1 ratio. A 50 μl medium-matrigel mix was added in 96-well plates on ice, and allowed to solidify in incubator (37 °C) before seeding the HUVECs. Each well was gently added with 15,000 HUVECs suspended in 50ul ECM medium without FBS and ECGS. Images were captured at 3 h after cell paved, and tube number was calculated with ImageJ software.

### Subcutaneous U87 Xenograft model

Animal experiments were approved by the Medical Ethics Committee of Yan’an People’s Hospital following the UK Animals (Scientific Procedures) Act, 1986 and Animal Research Reporting In Vivo Experiments (ARRIVE) guidelines. Six-week-old female BALB/c nude mice were purchased from GemPharmatech company (Nanjing, China). A total of 1 × 10^6^ control or ALKBH5 knock-down U87 GBM cells were resuspended in 200μL PBS (pH = 7.4) and injected into the backs of nude mice subcutaneously. The tumor size was measured every five days for each mouse when tumor growing. The nude mice were euthanized at the 20th day, and then the tumors were harvested and measured.

### Immunohistochemical (IHC) staining

The procedure of tumor sample IHC staining was performed, and the quantification was calculated according to a previous publication strictly^22^.

### Statistical analysis

Statistical analysis used in this study were conducted under R programming (version 4.1.3). The data with non-normal distribution between two groups were compared by Wilcoxon rank-sum test. As for normal distributed data, two-sided Student’s t-test was carried out. The protein expression of ALKBH5 in clinical samples were quantified and compared by two-sided paired t-test. Statistical correlations between two genes were quantified using Pearson Correlation analysis by R programming language.

### Ethics approval and consent to participate

This study is approved by the Medical Ethics Committee of Yan’an People’s Hospital, the usage of clinical samples and all methods were in strict accordance with the guideline of the Declaration of Helsinki. All enrolled inpatients have informed consent to participate in this study.

## Results

### Examining RNA modification regulators linked to angiogenesis

**Figure 1:**
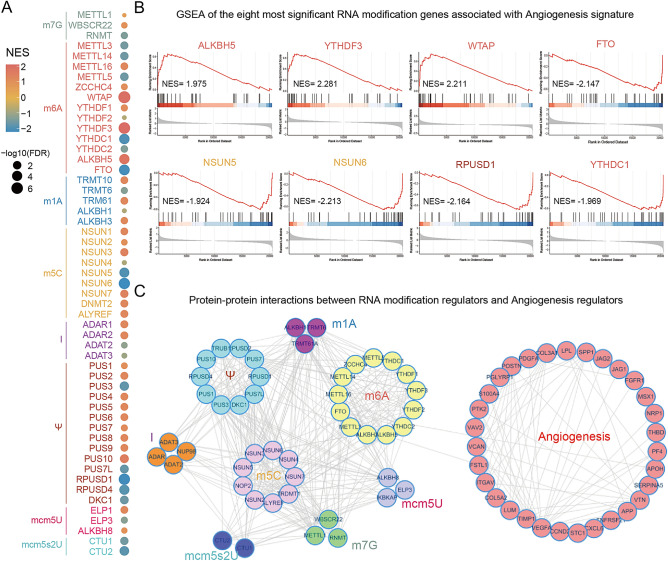
Screening of the angiogenesis associated RNA modification regulators in GBMs. (**A**) The bubble plot indicated the enriched level of each RNA modification regulators in GBM angiogenesis. (**B**) The top positive and negative enriched RNA modification regulators in GBM angiogenesis, including ALKBH5, YTHDF3 and WTAP, FTO, NSUN5, NSUN6, RPUSD1 and YTHDC1. (**C**) Protein–protein interaction (PPI) analysis of RNA modification gene coding proteins and angiogenesis regulator coding proteins.

The role of RNA modification in the angiogenesis of GBM microenvironment remains unclear, to address this, we employed the GSEA method to identify angiogenesis-related RNA modification (RM) regulators. A total of 53 RM regulators spanning eight types of RNA modification were subjected to GSEA for the Angiogenesis hallmark, the Fig. 1A shows the GSEA results of all RM regulators through a bubble plot. The color indicates the normalized enrichment score (NES) and the bubble size represents the false discovery rate (FDR) of the GSEA results. Subsequently, significant RM regulators were individually visualized based on NES values exceeding 1.9 or falling below − 1.9 (Fig. 1B). Notably, ALKBH5, YTHDF3, WTAP exhibited positive associations with the angiogenesis hallmark, while FTO, NSUN5, NSUN6, RPUSD1 and YTHDC1 displayed negative associations. To unravel the interaction landscape of proteins between RNA modification and angiogenesis, we conducted an analysis using STRING database (Fig. 1C). The results indicated fewer interactions or predictions between RM and angiogenesis regulators, emphasizing transcriptomic interactions as the primary means of linking RNA modification and angiogenesis in GBM.

### Correlations between angiogenesis-associated regulators and RNA modification regulators

**Figure 2:**
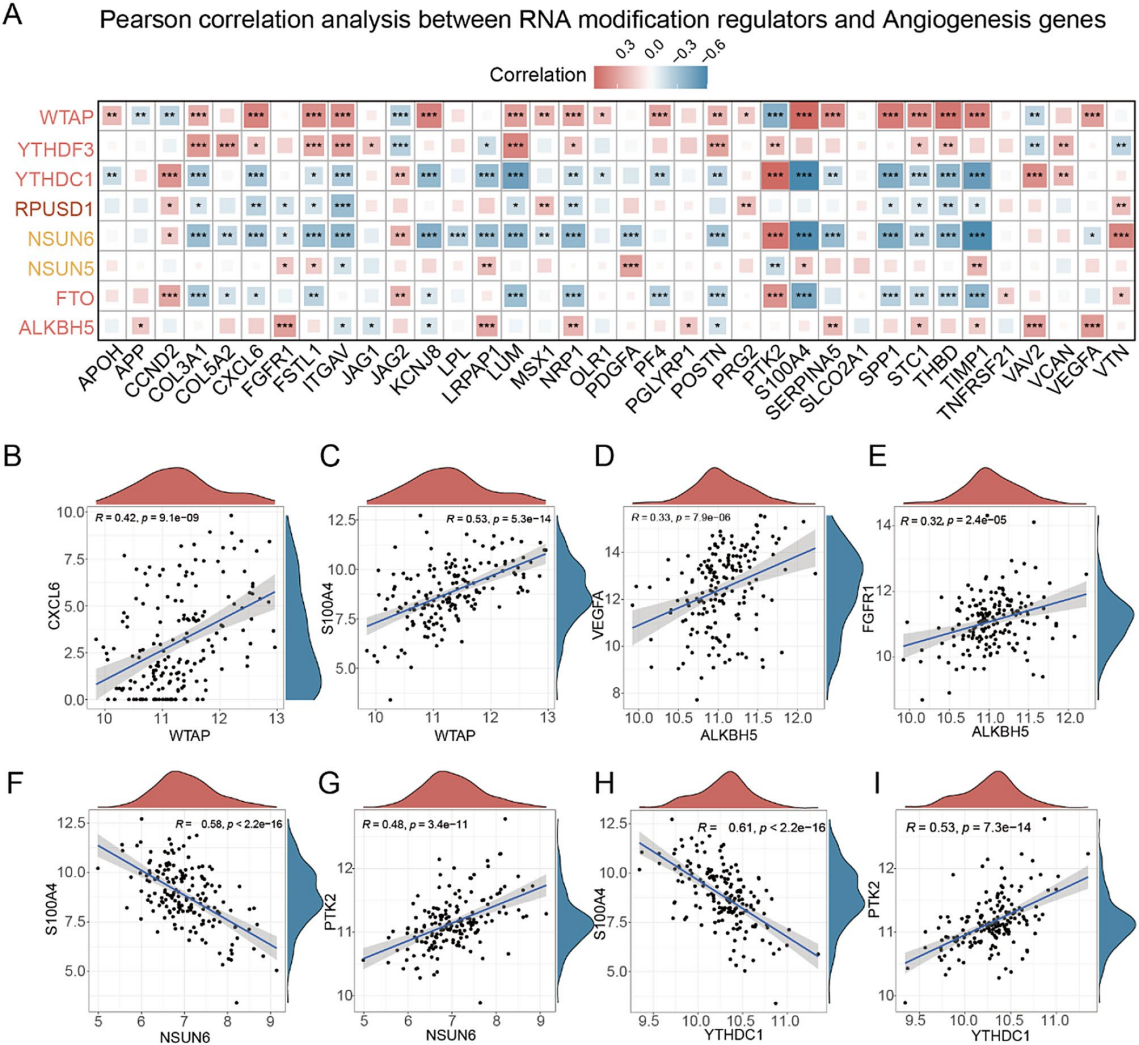
Correlation analysis between angiogenesis-associated RNA modification regulator and angiogenesis regulators. (**A**) Pearson correlation analysis between RNA modification regulators and angiogenesis genes in GBMs. (**B**) The top two significant angiogenesis regulators with WTAP, ALKBH5, NSUN8 and YTHDC1 in GBMs.

Thus, transcriptomic relationships between RM and angiogenesis regulators were investigated subsequently. The eight angiogenesis related RM regulators were selected to perform Pearson correlation analysis with 36 angiogenesis regulators using their transcriptomic data in GBM. The correlation results were visually represented in a heatmap, with red indicating positive correlations and blue indicating negative correlations (Fig. 2A). We visualized several significant correlations in scatter plots. Scatter plots illustrated significant correlations; for instance, WTAP exhibited positive associations with angiogenesis regulators like CXCL6 (Fig. 2B, Pearson R = 0.42, *p* < 0.0001) and S100A4 (Fig. 2C, Pearson R = 0.53, *p* < 0.0001). ALKBH5, as a m6A demethylase coding gene, showed positive correlations with VEGFA (Fig. 2D, Pearson R = 0.33, *p* < 0.0001) and FGFR1 (Fig. 2E, Pearson R = 0.32, *p* < 0.0001) expression. In contrast, NSUN6 displayed negative correlations with most angiogenesis regulators like S100A4 (Fig. 2F, Pearson R = 0.58, *p* < 0.0001) but positively associated with PTK2 expression (Fig. 2G, Pearson R = 0.48, *p* < 0.0001). YTHDC1 is also negatively associated with most angiogenesis regulators, indicating a broad relationship with the RNA expressions of angiogenesis regulators. These results indicated RNA modification regulators have a wide range relationship with angiogenesis regulators’ RNA expressions, which suggested they might regulate GBM angiogenesis via influencing RNA metabolism of angiogenesis regulators.

### Transcriptomic expression of RNA modification regulators in GBM

**Figure 3:**
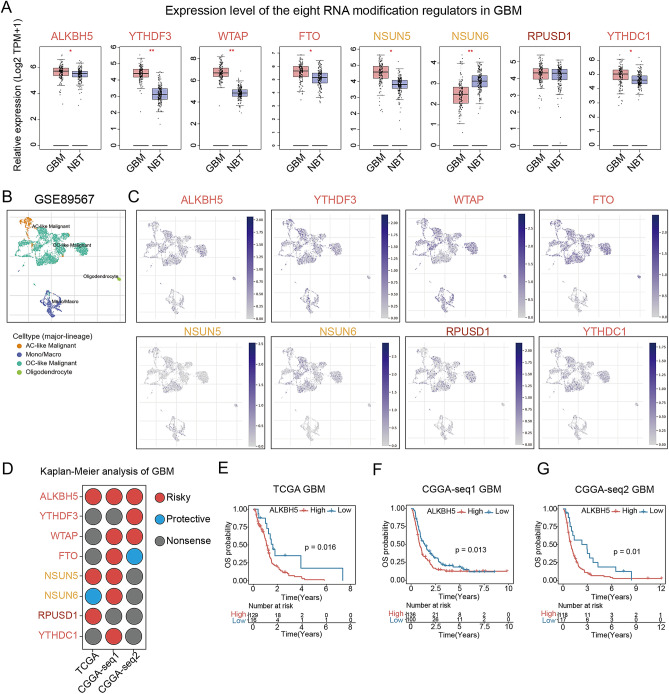
The aberrant expression, single-cell expression and prognostic role of the eight RNA modification regulators in GBMs. (**A**) The expression levels of the eight RNA modification regulator between GBM and normal brain tissues (NBTs), and results showed that ALKBH5, YTHDF3, WTAP, FTO, NSUN5, YTHDC1 were upregulated and NSUN6 was downregulated in GBMs. (**B**) The single cell RNA dataset GSE89567 contains four types of cells like: AC-like Malignant cells, Monocyte/Macrophages, OC-like Malignant cells and Oligodendrocytes. (**C**) The dot plots showed the expression distribution of each RNA modification regulators in GSE89567 dataset. (**D**) Three independent GBM cohorts (TCGA, CGGA-seq1 and CGGA-seq2) showed the prognostic roles of each RNA 507 modification regulators. (**E**,**F**) The Kaplan–Meier analysis showed the ALKBH5 is a risky and stable prognostic role in GBMs.

Our investigation delved into the roles of eight angiogenesis-associated RM regulators in GBMs. We compared the expressions of these regulators by integrating RNA-seq data from normal brain tissues (NBTs, GTEx database) and GBMs (TCGA database). As represented in Fig. 3A, ALKBH5, YTHDF3, WTAP, FTO, NSUN5 and YTHDC1 are significantly upregulated in GBMs, while NSUN6 decreases. Only RPUSD1 showed nonsense between NBTs and GBMs. To gain a detailed view of these RM regulators' expression distributions in GBM microenvironment, single cell analysis was conducted using a public GBM single-cell dataset GSE89567. The cells in GBM microenvironment are mainly divided into four types: AC-like Malignant, OC-like Malignant, oligodendrocyte and Mono/Macro cells (Fig. 3B). Across the eight RM regulators, ALKBH5, FTO, NSUN6, RPUSD1 and YTHDC1 were mainly expressed in malignant cells, while YTHDF3 and WTAP were highly expressed in both malignancy and monocytes/macrophages (Fig. 3C).

### Prognostic of angiogenesis-associated RNA modification regulators in GBMs

To access the clinical relevance of the eight RM regulators, we performed Kaplan–Meier survival analysis on three independent GBM cohorts (Fig. 3D). As shown in the survival analysis, only ALKBH5 acts as a risky factor in all the three GBM cohorts, the survival curves indicate that GBM patients with higher ALKBH5 expressions had a shorter survival time and rate (Fig. 3E–G). This part of results highlight ALKBH5 is not only associated with GBM angiogenesis, but also a stable prognostic biomarker for predicting GBM prognosis.

### Landscape of ALKBH5 protein in GBM

**Figure 4:**
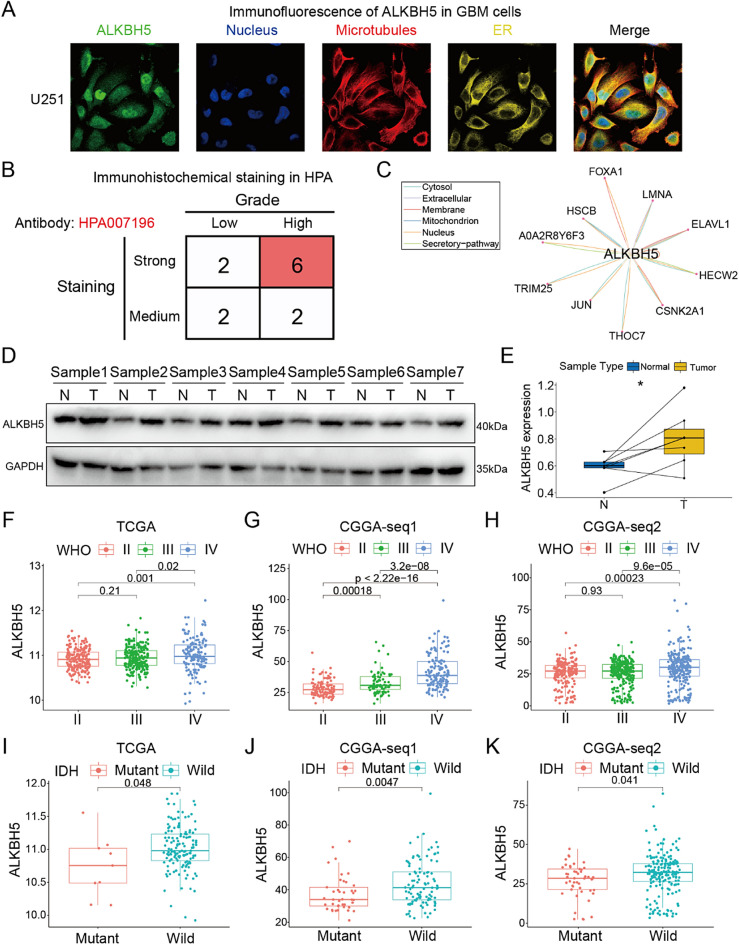
The protein information of ALKBH5 in GBMs. (**A**) The immunofluorescence showed the protein distribution of the subcellular of GBM cells. (**B**) The immunohistochemistry data of GBM samples in Human Protein Atlas (HPA) showed the ALKBH5 protein expression was strongly expressed in high-grade gliomas. (**C**) The protein–protein interaction analysis showed the ALKBH5 interactors. (**D**,**E**) Immunoblot suggested the expression of ALKBH5 protein in seven paired clinical GBM samples compared with adjacent tissues. (**F**–**H**) ALKBH5 expression level is highest in WHO grade IV glioma compared with WHO grade II/III gliomas in three public datasets. (**I**–**K**) ALKBH5 expression is higher in IDH-wild GBM samples than IDH-mutant GBMs.

Summarizing it all together, ALKBH5 emerges as an angiogenesis associated RM regulator, exhibiting higher expression in GBM tissues and cells, and serving as a predictor of GBM patient prognosis. Further exploration of ALKBH5's protein distribution, interactors, and expression in GBMs reveals its presence in both the nucleus and cytoplasm of U251 GBM cells (Fig. 4A) Immunohistochemistry staining images from the HPA datasets suggest higher expression of ALKBH5 in higher-grade gliomas (Fig. 4B). The protein–protein interaction (PPI) analysis identifies interactors such as FOXA1, LMNA, CSNK2A1, JUN, TRIM25, ELAVL1, HSCB, and HECW2, known for their crucial roles in GBM^23–27^ (Fig. 4C). Thus, the interactions between ALKBH5 and its interactors are also worthy to be paid attentions. Finally, the protein expression level was also accessed in our clinical GBM samples compared with adjacent tissues by western blotting (Fig. 4D, Original gel images showed in Supplementary Fig. 1A, B). Paired t-test was applied to access the different expression levels of ALKBH5 protein, and it indicated that ALKBH5 protein is significantly upregulated in GBM samples compared with NBTs (Fig. 4E). We also analyzed the ALKBH5 expression across gliomas with different WHO grades in the TCGA and CGGA cohorts, all the results indicated the highest expression of ALKBH5 in WHO IV gliomas (Fig. 4F–H). Besides, a higher ALKBH5 expression is observed in IDH-wild GBM compared IDH-mutant GBM (Fig. 4I–K). These results underscore the association between ALKBH5 and glioma malignant phenotypes.

### Gene ontology (GO) and Kyoto encyclopedia of genes and genomes (KEGG) enrichment analysis of ALKBH5 in GBM

**Figure 5:**
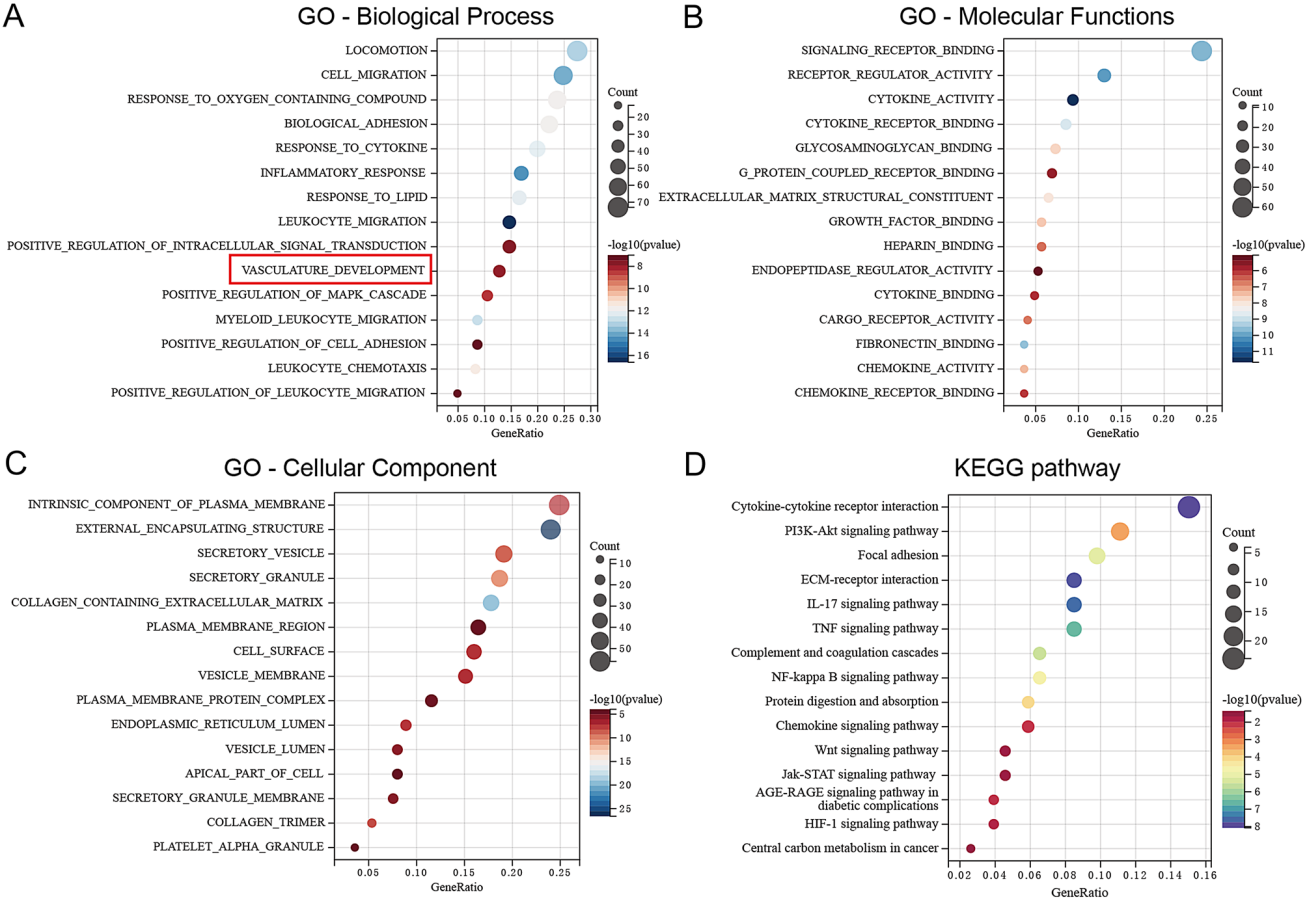
Enrichment analysis of ALKBH5 related DEGs in GBMs. The bubble plots represent the enriched biological processes. (**A**) Molecular functions (**B**) cellular components (**C**) and KEGG pathways (**D**) respectively.

To elucidate the biological processes, molecular function, cellular component and pathway associations of ALKBH5 in GBMs, we conducted gene ontology (GO) and Kyoto Encyclopedia of Genes and Genomes (KEGG) analysis in GBM by categorizing GBMs into ALKBH5-low and ALKBH5-high two subgroups. In the most significant biological process, vasculature development was enriched in ALKBH5-high GBMs (Fig. 5A), which is consistent with our GSEA analysis. Additionally, ALKBH5-high GBMs exhibited enrichment in cell migration, biological adhesion, inflammatory and response. Cytokine-associated activities were significantly enriched in ALKBH5-high GBMs in terms of molecular functions (Fig. 5B). Regarding cellular components, secretory associated cellular components were significantly enriched in ALKBH5-high GBMs (Fig. 5C). The most interesting is that the enriched KEGG pathways in ALKBH5-high GBMs include PI3K-AKT signaling, TNF signaling, NF-κB signaling, HIF-1 signaling pathways (Fig. 5D), all of these oncogenic pathways had been reported to have angiogenesis promotive functions in GBMs.

### ALKBH5 Konck-down impairs the pro-angiogenesis ability of U87 cells in vitro

**Figure 6:**
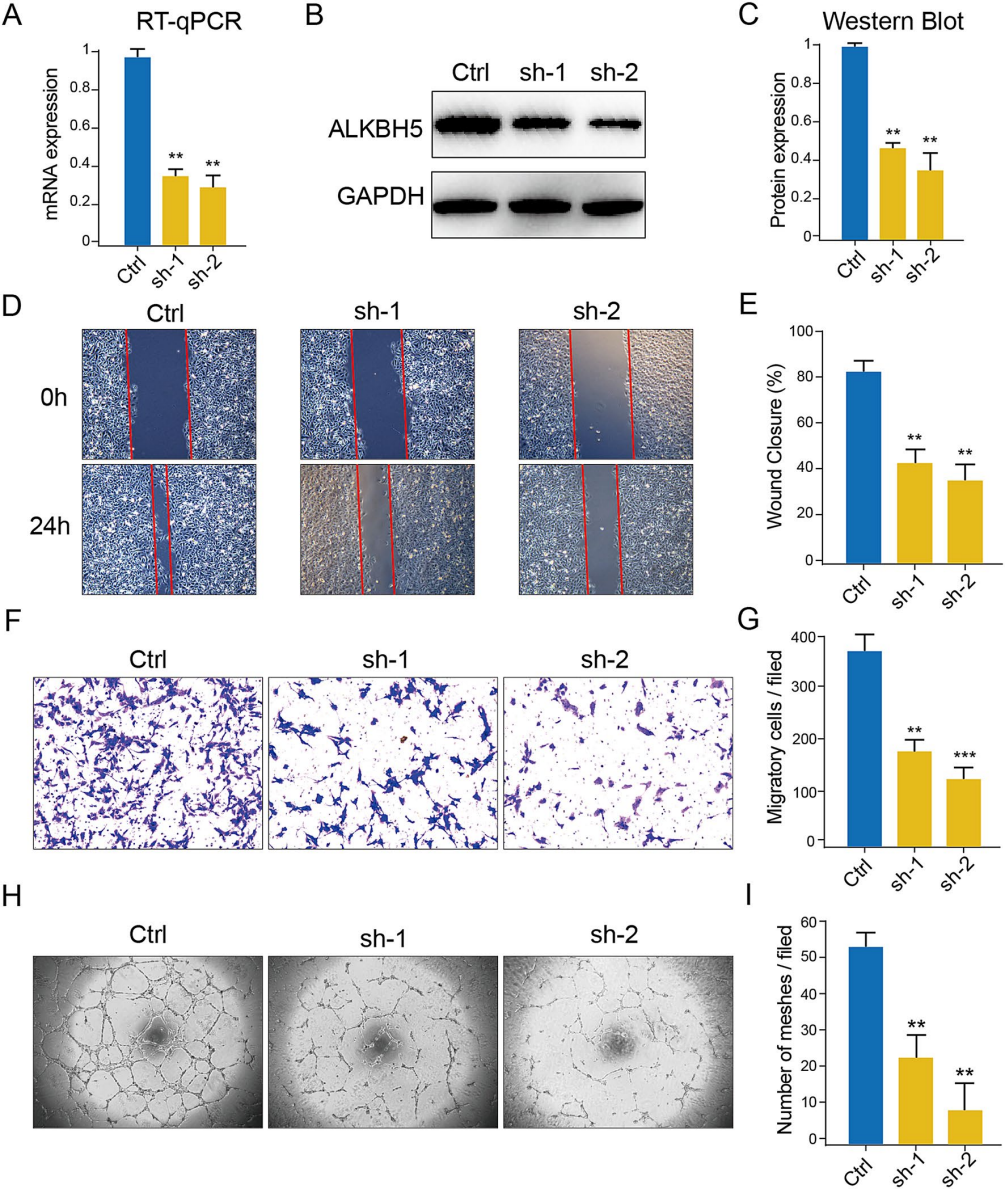
Downregulation of ALKBH5 in GBM cells impairs the angiogenesis ability of co-cultured HUVECs in vitro. (**A**–**C**) RT-qPCR (**A**) and Western blot (**B**,**C**) assays showed the knock-down efficacy of shRNAs in U87 GBM cells. (**D**–**G**) Wound healing (**D**,**E**) and transwell (**F**,**G**) assays showed knock-down of ALKBH5 in GBM cells impaired the migration ability of co-cultured HUVECs in vitro. (**H**–**I**) Tube formation assay represents knock-down of ALKBH5 in GBM cells suppress the tube formation capacity of co-cultured HUVECs in vitro.

To access the role of intrinsic ALKBH5 in GBM angiogenesis, two shRNA sequences targeting ALKBH5 from a previous study^28^ were used to downregulate the expression of ALKBH5. Both RT-qPCR and western blot confirmed the successful downregulation of ALKBH5 by both shRNAs (Fig. 6A-C, Original gel images showed in Supplementary Fig. 2A, B). Subsequent assays demonstrated that the culture medium from sh-ALKBH5-U87 cells reduced the migratory ability of co-cultured HUVECs in wound healing and transwell assays compared to control U87 cells (Fig. 6D–G). This indicated that down-regulation of ALKBH5 in GBM cells could influence the mobility of HUVEC via the method of external secretion. To be sure of the impact of ALKBH5 in angiogenesis, Tube formation assays also revealed that ALKBH5 downregulation in U87 cells impacted the angiogenic potential of co-cultured HUVECs (Fig. 6H,I).

### Down-regulation of ALKBH5 decreases VEGFA expression in vitro and in vivo

**Figure 7:**
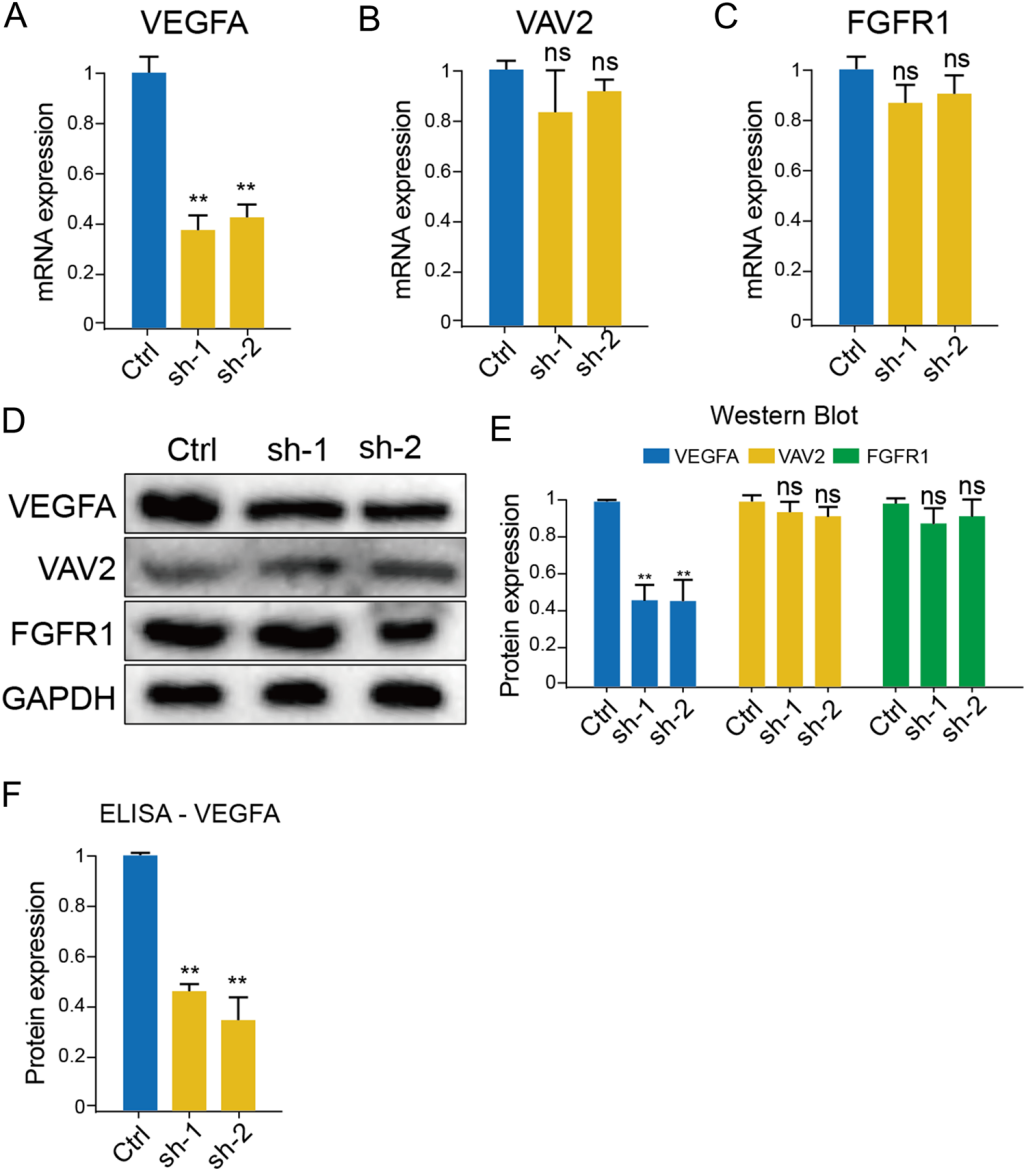
Downregulation of ALKBH5 in U87 cells can reduce VEGFA expression and secretion. (**A–C**) Knock-down ALKBH5 can reduce VEGFA (**A**) mRNA expression in U87 GBM cells, rather than VAV2 (**B**) and FGFR1 (**C**). (**D**,**E**) Western blot analysis also proved the protein expression of VEGFA were decreased in sh-ALKBH5 U87 cells. (**F**) Knock-down ALKBH5 in U87 cells also impaired the secretion of VEGFA.

To elucidate how ALKBH5 regulats GBM angiogenesis, we selected FGFR1, VAV2 and VEGFA as potential downstream angiogenesis regulator according to the bioinformatic results (Fig. 2A), and verified the expression levels of FGFR1, VAV2 and VEGFA in ALKBH5-knock-down U87 cells. By RT-qPCR analysis, we found VEGFA mRNA expressions were downregulated in sh-ALKBH5 U87 cells (Fig. 7A), rather than VAV2 and FGFR1 (Fig. 7B,C). Besides, western blot also verified the VEGFA downregulation effect in sh-ALKBH5 U87 cells (Fig. 7D,E, Original images showed in Supplementary Fig. 3A–D). Finally, considering VEGFA is secreted to promote cancer angiogenesis, we also test the VEGFA levels in the culture medium of sh-ALKBH5 U87 cells (Fig. 7F), and results also indicated knock-down ALKBH5 in U87 cells will lead to less VEGFA expression and secretion.

**Figure 8:**
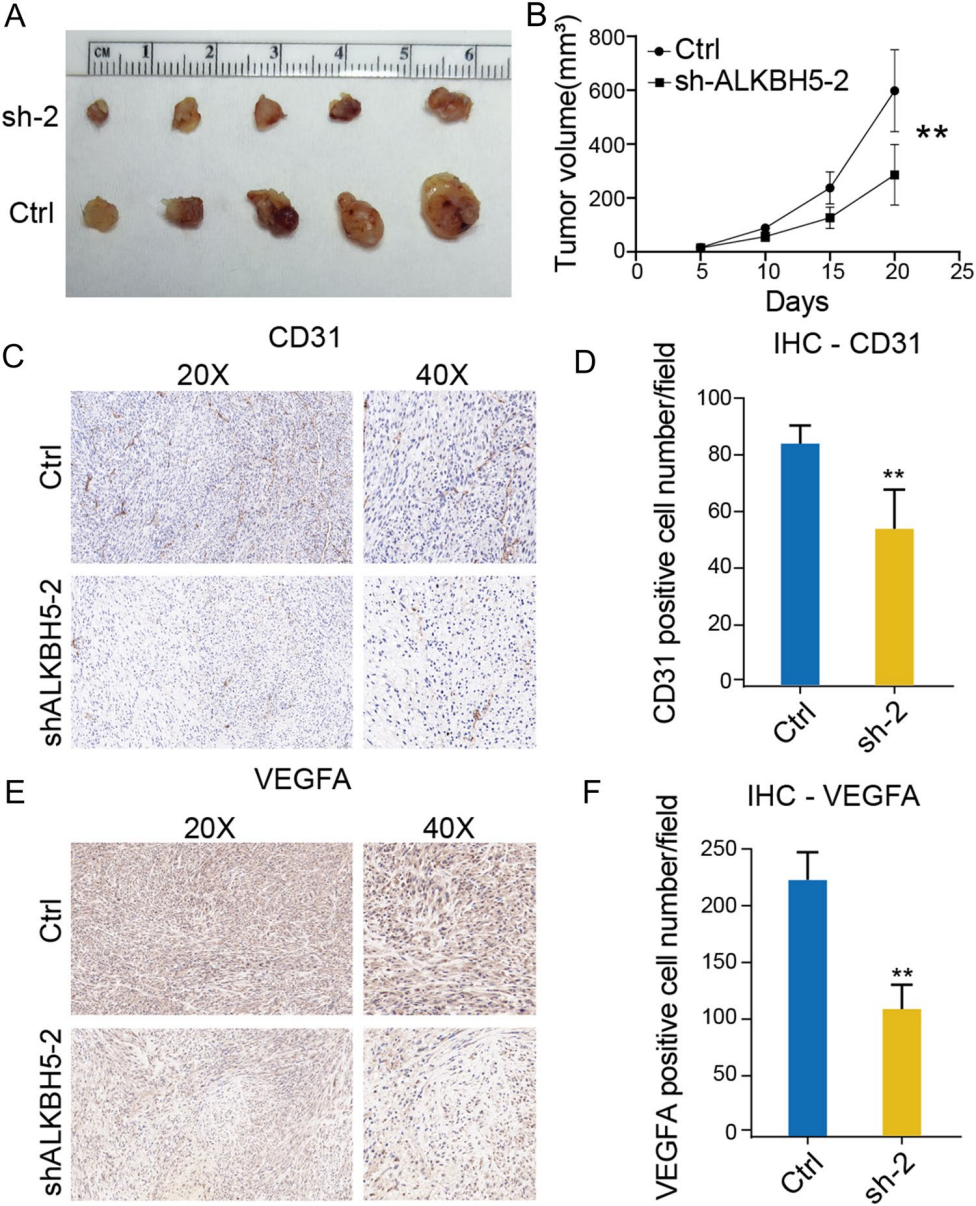
Inhibition of ALKBH5 in U87 xenograft significantly reduce tumor angiogenesis. (**A**,**B**) The sh-ALKBH5 U87 subcutaneous tumors showed smaller size compared with control tumors. (**C**,**D**) sh-ALKBH5 U87 tumor showed lower staining of CD31 compared with control U87 tumors. (**E**,**F**) sh-ALKBH5 U87 tumor showed lower staining of VGFFA compared with control U87 tumors.

In vivo experiments using subcutaneous implantation of sh-Con and sh-ALKBH5 U87 cells in nude mice demonstrated a significant decrease in tumor size with ALKBH5 knockdown (Fig. 8A,B). And by detecting VEGFA and CD31 expressions in tumors via immunohistochemical (IHC) staining, we proved that both VEGFA (Fig. 8C,D) and CD31 (Fig. 8E,F) were downregulated in sh-ALKBH5 U87 subcutaneous tumors, which means angiogenesis activities were impaired in sh-ALKBH5 U87 subcutaneous tumors. These findings collectively indicate that ALKBH5 plays a crucial role in promoting GBM angiogenesis through modulation of VEGFA expression.

## Discussion

RNA modification has become a focal point in cancer research, and while the role of ALKBH5 has been established in regulating endothelial cell angiogenesis via a SPHK1 dependent manner under ischemic stress, but the crosstalk between RNA modification and angiogenesis of GBM has been less explored. This comprehensive study systematically gathered RNA modification regulators and GBM clinical cohorts, employing various bioinformatic methods to uncover potential associations with the angiogenesis hallmark in GBMs. ALKBH5 is identified as a noteworthy RNA modification regulator linked to angiogenesis, exhibiting a significant positive association with VEGFA expression—core regulator in GBM angiogenesis. Besides, it also showed a stable and promising prognostic role in predicting the prognostic clinical GBM patients. In vitro and in vivo experiments further validated the role of intrinsic ALKBH5 in GBM angiogenesis via regulating VEGFA expression and secretion.

The study employed a heuristic approach, employing GSEA, differential expression, and prognostic analysis to systematically identify hallmark-correlated RNA modification regulators, with ALKBH5 emerging as a novel player in GBM angiogenesis regulation. While ALKBH5 has been previously implicated in GBM invasion, radio-resistance, chemo-resistance and microenvironment shaping^29–31^, this study is the first to establish its role in angiogenesis regulation. Considering the vital function of ALKBH5 in mRNA biology, it is speculated that ALKBH5 may control the angiogenesis via direct RNA modification method or indirectly way, this point needs subsequent experiments to understand.

Despite demonstrating ALKBH5's pro-angiogenesis role in GBM and establishing that down-regulation of ALKBH5 in GBM substantially reduces both in vitro and in vivo VEGFA expression and secretion, there remain several limitations that must be addressed. The first is the unclear deeper mechanism, which requires further investigation. The second point concerns the translation of research findings into clinical applications, specifically the need for additional testing to determine whether ALKBH5 inhibitors can effectively restrain GBM angiogenesis.

A recent publication found ALKBH5 can facilitate the advancement and angiogenesis of lung cancer by modulating the stability of the long non-coding RNA (LncRNA) PVT1^32^, and PVT1 was also reported highly upregulated in GBM tissues and cells, and involved in GBM malignant progression^33^. This potential mechanism identified in ALKBH5-regulated GBM angiogenesis suggests a promising direction for further exploration in pursuit of our research objectives.

In summary, these findings present a fresh opportunity to tackle GBM by inhibiting angiogenesis through the suppression of RNA modification, specifically targeting ALKBH5. While the present results are promising, the subsequent stages entail conducting comprehensive analyses and translating these theoretical insights into tangible applications for potential therapeutic interventions.

### Supplementary Information

Supplementary Figures.

## Data Availability

The data used in this study were public datasets, and could be obtained from the websites listed below: UCSC (TCGA-GBM cohort ,https://xenabrowser.net/datapages/), CGGA (mRNAseq_693 and mRNAseq_325 cohorts, http://www.cgga.org.cn/), GEO (GSE89567, https://www.ncbi.nlm.nih.gov/gds), GTEx (file = ”GTEx_Analysis_2017-06-05_v8_RNASeQCv1.1.9_gene_tpm.gct.gz”, https://gtexportal.org/home/).

## Abbreviations

GBM: Glioblastoma
GSEA: Gene set enrichment analysis
m6A: N6-methyladenosine
GO: Gene ontology
KEGG: Kyoto encyclopedia of genes and genomes
HUVEC: Human umbilical vein endothelial cell
OS: Overall survival
VEGF: Vascular endothelial growth factor
m7G: N7-methylguanosine
m1A: N1-methyladenosine
m5C: 5-Methylcytidine
mcm5U: 5-Methoxycarbonylmethyluridine
I: Adenosine to inosine transition
ψ: Pseudouridine
PPI: Protein–protein interaction
GTEx: Genotype-tissue expression
DEGs: Differential expression genes
NES: Normalized enrichment score
NBT: Normal brain tissue
GEO: Gene expression omnibus
HPA: Human protein atlas
BP: Biological process
MF: Molecular function
CC: Cellular component
