# Supplementary material for: ALKBH5 is a prognostic factor and promotes the angiogenesis of glioblastoma
Source: Sci Rep. 2024 Jan 14;14:1303. doi: 10.1038/s41598-024-51994-9 (PMC10788339; doi:10.1038/s41598-024-51994-9)
Supplement: Supplementary file 1 — Supplementary Figures. [file 41598_2024_51994_MOESM1_ESM.pdf]

A

ALKBH5

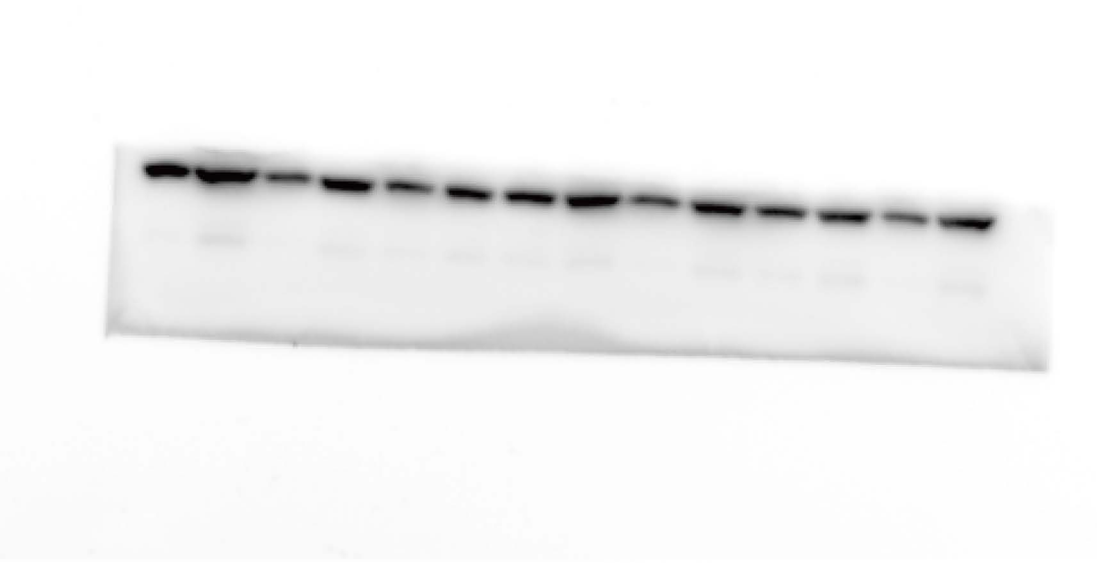

B

GAPDH

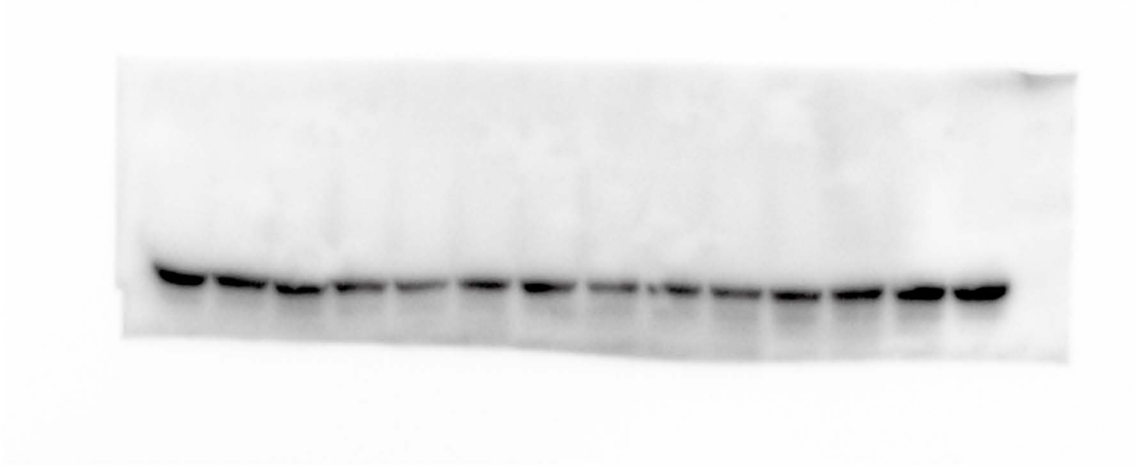

Supplementary Figure1: The orignal gel of clinical samples represent the ALKBH5 (A) and GAPDH (B) expressions.

A

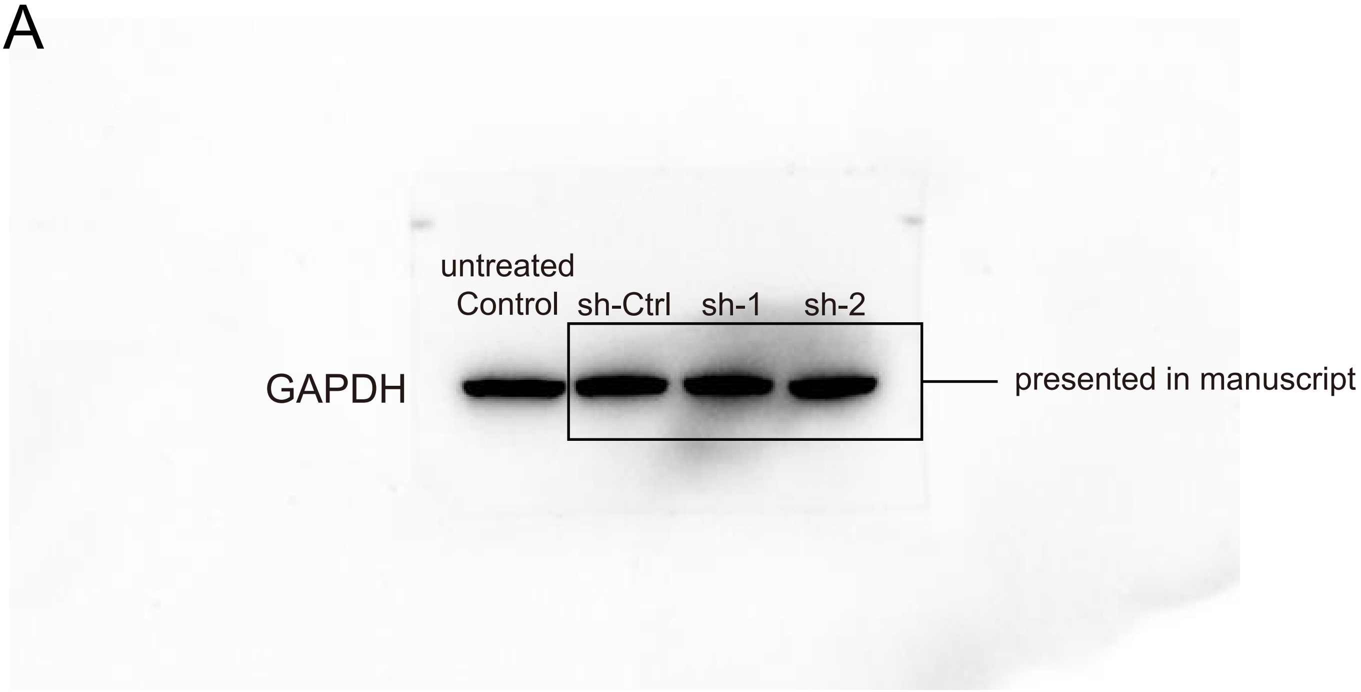

B

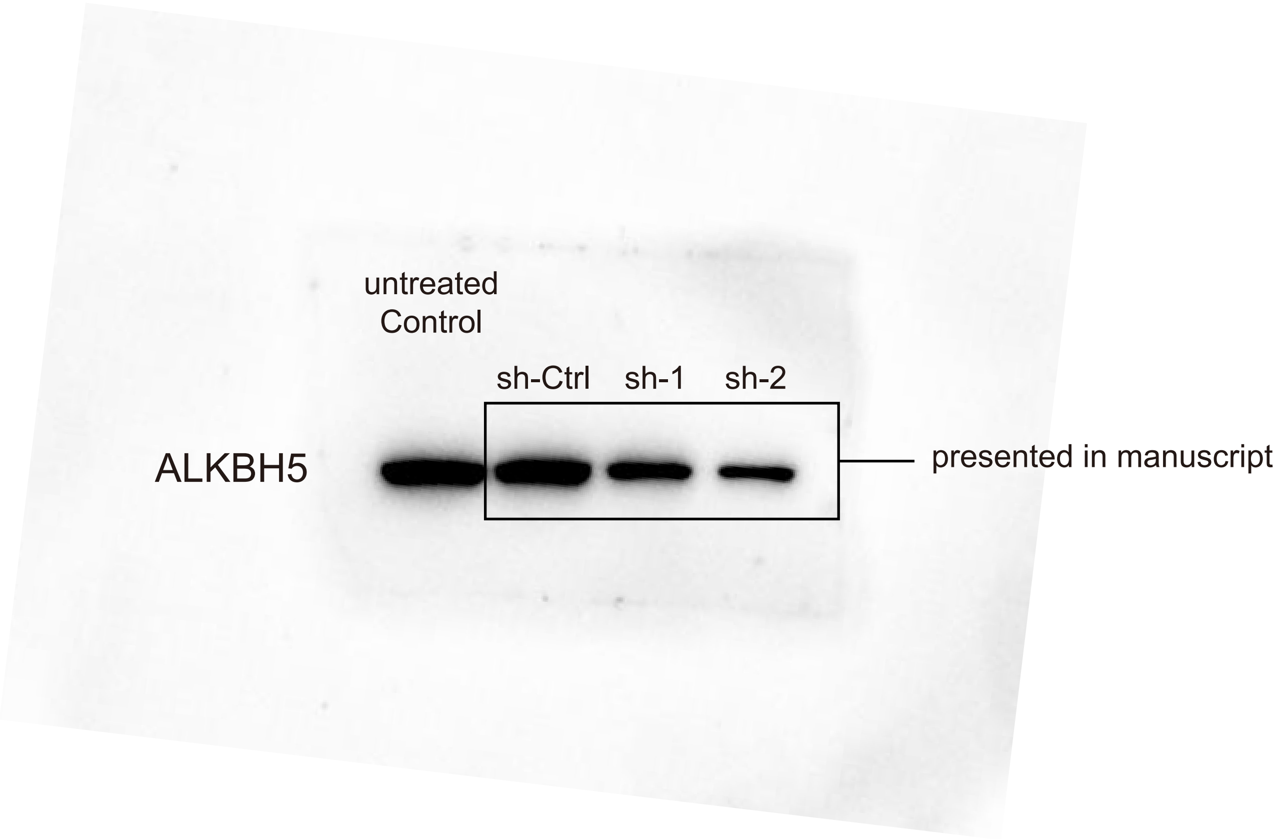

Supplementary Figure2: The orignal gel of U87 cells transfected with shRNAs represent the ALKBH5 (B) and GAPDH (A) expressions.

A

VEGFA

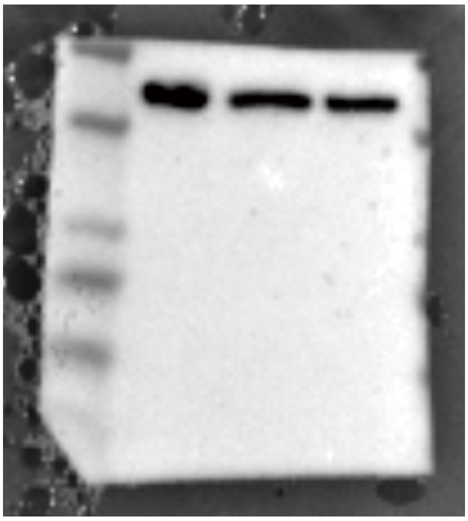

B

FGFR1

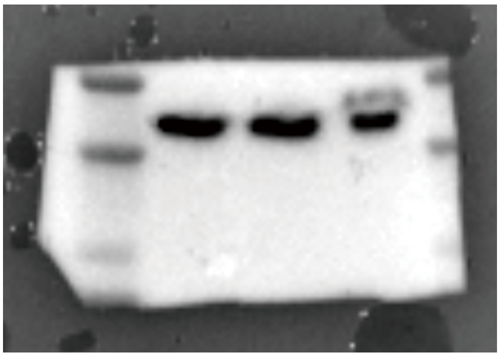

C

VAV1

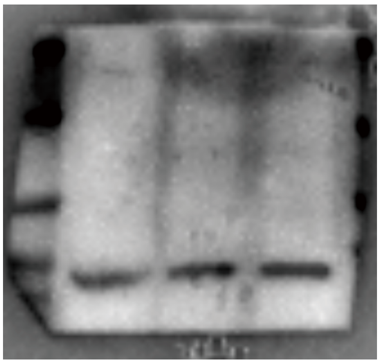

D

GAPDH

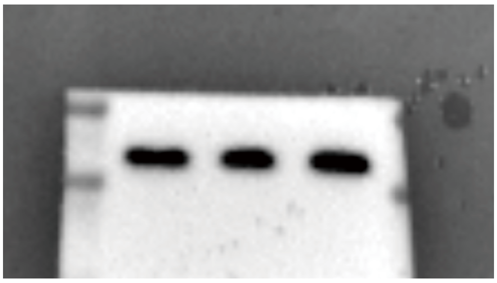

Supplementary Figure3: The orignal gel of U87 cells transfected with shRNAs represent the VEGFA (A),FGFR1(B), VAV1(C) and GAPDH (D) expressions.
